# Exploratory and Confirmatory Factor Analysis of the 9-Item Utrecht Work Engagement Scale in a Multi-Occupational Female Sample: A Cross-Sectional Study

**DOI:** 10.3389/fpsyg.2019.02771

**Published:** 2019-12-06

**Authors:** Mikaela Willmer, Josefin Westerberg Jacobson, Magnus Lindberg

**Affiliations:** ^1^Department of Health and Caring Sciences, Faculty of Health and Occupational Studies, University of Gävle, Gävle, Sweden; ^2^Department of Public Health and Caring Sciences, Uppsala University, Uppsala, Sweden

**Keywords:** confirmatory factor analysis, exploratory factor analysis, Utrecht work engagement scale, work engagement, occupational psychology

## Abstract

**Objective:**

The aim of the present study was to use exploratory and confirmatory factor analysis (CFA) to investigate the factorial structure of the 9-item Utrecht work engagement scale (UWES-9) in a multi-occupational female sample.

**Methods:**

A total of 702 women, originally recruited as a general population of 7–15-year-old girls in 1995 for a longitudinal study, completed the UWES-9. Exploratory factor analysis (EFA) was performed on half the sample, and CFA on the other half.

**Results:**

Exploratory factor analysis showed that a one-factor structure best fit the data. CFA with three different models (one-factor, two-factor, and three-factor) was then conducted. Goodness-of-fit statistics showed poor fit for all three models, with RMSEA never going lower than 0.166.

**Conclusion:**

Despite indication from exploratory factor analysis (EFA) that a one-factor structure seemed to fit the data, we were unable to find good model fit for a one-, two-, or three-factor model using CFA. As previous studies have also failed to reach conclusive results on the optimal factor structure for the UWES-9, further research is needed in order to disentangle the possible effects of gender, nationality and occupation on work engagement.

## Introduction

Work engagement has been described as the conceptual opposite of burnout (González-Romá et al., 2006), and as such belongs in the area of positive psychology, or “the study of the conditions and processes that contribute to the flourishing or optimal functioning of people, groups, and institutions”(Gable and Haidt, 2005). In occupational health, the study of work engagement focuses on factors that contribute to job satisfaction as well as long-term mental and physical health (Torp et al., 2013).

Work engagement has been described as “a positive work-related state of mind characterized by vigor, dedication and absorption.” (Schaufeli et al., 2002). These three concepts are in their turn described as “characterized by high levels of energy and mental resilience while working, the willingness to invest effort in one’s work, and persistence even in the face of difficulties” (Vigor), “characterized by a sense of significance, enthusiasm, inspiration, pride and challenge” (Dedication) and “characterized by being fully engrossed in one’s work, so that time passes quickly and one has difficulties in detaching oneself from work” (Absorption) (Schaufeli et al., 2002).

The idea that these three concepts – Vigor, Dedication and Absorption – together form the foundation of work engagement forms the basis of the Utrecht work engagement scale (UWES) (Schaufeli et al., 2002). Originally a 17-item questionnaire (UWES-17), the original authors have shortened it to a 9-item version (UWES-9) in order to reduce the burden on the respondents and minimize attrition (Schaufeli et al., 2006). The items are in the form of statements (for example “At my work, I feel bursting with energy” (Vigor); “I find the work that I do full of meaning and purpose” (Dedication); “When I am working, I forget everything else around me” (Absorption) which the respondent reads and reacts to by indicating one of 7 points on a scale ranging from 0 (“Never”) to 6 (“All the time”). The 9-item version, which has been psychometrically tested in various countries and samples (Ho Kim et al., 2017; Petrović et al., 2017), will be the focus of the present study.

In a number of studies, conducted in different countries and with samples of various make-ups, UWES-9 scores have been found to be associated with work performance, job satisfaction, and mental and physical health (Bakker and Matthijs Bal, 2010; Christian et al., 2011). The scores have also been found to predict general life satisfaction and the frequency of sickness absence (Leijten et al., 2015).

Despite its wide-spread use, both the UWES-17 and the UWES-9 have been the subject of some criticism. Mills et al. (2012) have argued that the methodology when developing the original scale contained flaws in relation to the establishment of its factorial structure. Criticism has also been voiced regarding the factor structure of the instrument, one of the main points being that the three subscales Vigor, Dedication and Absorption are very closely correlated with each other, casting doubt on the three-factor structure’s superiority to a one-factor structure using only the total score on the scale (Kulikowski, 2017). For example, Shirom has argued that the three dimensions of Vigor, Dedication, and Absorption were not theoretically deduced and that they overlap each other conceptually (Shirom, 2003). In support of this, several studies have failed to confirm the three-factor structure in their samples. Previous studies have also tested other factor structures – for example, Kulikowski (2019) tested a two-factor structure, with Dedication and Vigor merged into a single factor and Absorption constituting a second factor (Kulikowski, 2019). A 2017 review by Kulikowski investigated the factorial structure of the UWES-17 and UWES-9 as reported in 21 different studies, conducted in 24 countries using samples from a variety of occupations and countries. The author found that of the 11 studies investigating the UWES-9, three confirmed the one-factor structure, three the three-factor structure, four studies found these two factor structures to be equivalent, and one study failed to support either alternative (Kulikowski, 2017). Thus, Kulikowski (2017) concluded that no definitive recommendations could be made based on the review. He also pointed out the importance, in light of these inconclusive results, that further research be conducted on the factorial structure of the UWES-9 in different samples (Kulikowski, 2017).

Only one previous study has tested the factorial validity of the UWES-9 in a Swedish sample (Hallberg and Schaufeli, 2006). In their sample of 186 information communication technology consultants (of whom 37% were women), both the one-factor and three-factor structures were supported by data, leading the authors to draw the conclusion that both options were equally strong. If the scope is broadened to take in all the Scandinavian countries, a Norwegian study using a large multi-occupational sample (*n* = 1266, 67% women) found support for the three-factor structure, but also found that the three latent factors were strongly correlated, leading the authors to suggest that a one-factor structure might also be suitable (Nerstad, Richardsen and Martinussen, 2010). In addition to this, a Finnish study found, in a sample of 9404 workers in several different occupational sectors, that both the one-factor and three-factor structures may reasonably be used (Seppälä et al., 2009). Similarly to the Norwegian study, the results showed that the three subscales of Vigor, Dedication, and Absorption were highly correlated.

Interestingly, it has been suggested that as a rule, levels of work engagement tend to be higher in countries in Northwestern Europe, and lower in Southern Europe, on the Balkans and in Turkey (Schaufeli, 2018). However, Sweden is identified as an exception to this rule, with relatively low levels of work engagement compared to, for example, Norway, where levels were found to be higher (Schaufeli, 2018).

The 9-item UWES is a widely used instrument to measure work engagement. Despite this, the optimal factorial structure of the UWES-9 remains unknown. A recent review of factorial structure for the UWES-9 and UWES-17 failed to reach conclusive results, and indicated that more research was needed to determine the appropriate default factorial structure (Kulikowski, 2017). Many previous studies have used relatively small samples, and many have reached inconclusive results, including the only previously published Swedish study. In order to adequately assess and potentially target work engagement in future interventions using Swedish populations, it is important to examine and ascertain whether Swedish people hold the same representation of work engagement. Thus, the aim of the present study was to use exploratory and confirmatory factor analysis (CFA) to investigate the factorial structure of the 9-item UWES in a multi-occupational Swedish sample.

## Materials and Methods

### Participants

The women in the all-female sample used for the current study were originally recruited in 1995, when they were aged between 7 and 15 years, through stratified randomization from a number of school classes in Sweden. They were sampled to represent a general population of girls, and were participants in a longitudinal study aiming to identify risk and protective factors for the development of eating disorders. More details about the recruitment and follow-up can be found elsewhere (Westerberg-Jacobson et al., 2010). The data used in the current study was collected in 2015, as part of the 20-year follow-up data collection. The participants remaining in the study were asked to complete a number of questionnaires, including the UWES-9, and those who indicated that they were currently working full-time or part-time (not on long-term sick-leave, parental leave, unemployed, or studying full-time) were included in the current study. Thus, the final sample consisted of 702 women, aged between 26 and 37, who completed a Swedish translation of the 9-item UWES (Schaufeli et al., 2006). Aside from the UWES-9, data was collected on level of education (primary school, secondary education or university education), although not on specific occupation.

### Ethics Statement

The project was approved by the Regional Ethics Board in Uppsala, Sweden (2014/401). At the time of the original recruitment, in 1995, the participants and their parents gave written informed consent to take part in the study. At the time of the data collection for the present study, the participants again gave their written informed consent and were reminded that their participation was voluntary, could be withdrawn any time without giving a reason, and that all information would be treated confidentially. All participants who completed the data collection were offered a cinema ticket or a department store gift voucher as thanks.

### Statistical Analysis

All analyses were performed using Stata 14 (StataCorp, 2015) and SPSS (IBM Corp, 2016) statistical software packages. The Kaiser-Meyer-Olkin Measure of Sampling Adequacy and Bartlett’s Test of Sphericity were used to assess the suitability of the data for factor analysis (Dziuban and Shirkey, 1974). Exploratory factor analysis (EFA) was first performed unrotated, using maximum likelihood extraction and eigenvalues > 1. Additionally, we performed EFA with promax rotation and enforcing three-factor solution in order to test the theoretical structure of the UWES-9. In this analysis, we also used maximum likelihood extraction. Additionally, Parallel Analysis (using principal axis factoring) and Velicer’s Minimum Average Partial test were conducted (O’Connor, 2000).

CFA was then performed using maximum likelihood estimation.

In order to investigate the models’ goodness of fit, a number of statistics were used: Overall χ^2^ (Hooper et al., 2008), root mean square error of approximation (RMSEA) (Steiger, 1990; Hooper et al., 2008), Akaike’s information criterion (AIC), Bayesian information criterion (BIC), comparative fit index (CFI), Tucker-lewis index (TLI) (Bentler, 1990), and the standardized root mean square residual (SRMSR) (Hooper et al., 2008).

## Results

Demographic information about the participants can be seen in Table 1. Data on highest attained educational level was collected, and showed that the majority of the sample had attended at least 3 years of higher education.

**TABLE 1:** Demographic information about the participants.

**Variable (*n* = 702)**		
	**Mean**	**Standard deviation**

Age	31.8 (2.9)	

**Marital status**	**Frequency**	**Percentage**

Single	159	23
Married/cohabiting	530	76
Divorced	9	1

**Education**	**Frequency**	**Percentage**

Compulsory (9 years)	9	1
<3 years upper secondary	21	3
≥3 years upper secondary	152	22
<2 years university	75	11
≥2 years university	425	61

**UWES scores**	**Mean**	**Standard deviation**

Total UWES score	4.06	1.18
Vigor	3.96	1.19
Dedication	4.24	1.25
Absorption	3.98	1.32

The inter-item correlation was relatively high for all items of the UWES-9, ranging between 0.524 and 0.849. The three subscales Vigor (V), Dedication (D), and Absorption (A) also showed high correlation with each other (0.79–0.84). In addition to this, Cronbach’s alpha was calculated and found to be 0.947, indicating very good internal consistency.

The items were checked for skewness and kurtosis and these are shown in Table 2, together with the wording of the items, their respective subscales, mean scores and standard deviations. Based on the Shapiro-Wilks test and a visual inspection of their histograms, normal Q-Q plots and box-plots, we concluded that the UWES item distributions had a skewness range between −0.560 and −1.262 (SE = 0.094) and a kurtosis range between −0.046 and 1.645 (SE = 0.187) (Table 2). The values for skewness and kurtosis were deemed to be within the range for maximum likelihood estimation. We also tested the multivariate normality using Doornik-Hansen test, the Mardia skewness test and Mardia kurtosis test. For all of these, the *p*-value was <0.0001, indicating non-normality.

**TABLE 2:** Items with their subscales, mean scores, standard deviations, skewness, and kurtosis.

**Item (subscale)**	**Mean**	**Standard deviation**	**Skewness**	**Kurtosis**
1. At my work, I feel bursting with energy (V)	3.93	1.30	−0.798	0.294
2. At my job, I feel strong and vigorous (V)	4.08	1.22	−0.921	0.678
3. I am enthusiastic about my job (D)	4.10	1.32	−0.900	0.568
4. My job inspires me (D)	4.01	1.44	−0.808	0.266
5. When I get up in the morning, I feel like going to work (V)	3.89	1.49	−0.805	0.113
6. I feel happy when I am working intensely (A)	3.89	1.49	−0.711	−0.046
7. I am proud of the work that I do (D)	4.62	1.32	−1.262	1.645
8. I get carried away when I am working (A)	4.43	1.31	−1.159	1.474
9. I am immersed in my work (A)	3.64	1.64	−0.560	−0.497

*V, vigor; D, dedication; A, absorption.*

In the next step, the sample was randomly divided in two, so that mutually independent samples were obtained for the EFA and CFA, respectively. As the number of participants with missing values was very low (19 individuals, corresponding to 3% of the entire sample), only observations without any missing items were used, resulting in 683 observations in total, 341 for the EFA and 342 for the CFA.

### Exploratory Factor Analysis

The results of the EFA suggested that one factor explained over 70% of the variance. The Kaiser-Meyer-Olkin Measure of Sampling Adequacy was 0.922, indicating that the sample was adequate, and Bartlett’s Test of Sphericity gave a *p*-value of <0.001. A Scree plot of the eigenvalues was constructed (not shown) and shown to be strongly in favor of the one-factor structure. The χ2 for this model was 332,43 (df 27).

Velicer’s MAP test was also performed, both in the original (Velicer, 1976) and revised version (O’Connor, 2000). This also strongly pointed toward a one-factor solution.

Finally, in the Parallel Analysis, the raw data eigenvalue from the actual data was greater than eigenvalues of the 95th percentile of the distribution of random data for four factors, in disagreement with the MAP test and the EFA (O’Connor, 2000).

Table 3 shows the factor loadings. As the table shows, all loadings were relatively high, ranging from 0.65 to 0.93.

**TABLE 3:** Factor loadings.

**Variable**	**Factor 1**
UWES1	0.78
UWES2	0.81
UWES3	0.93
UWES4	0.90
UWES5	0.81
UWES6	0.86
UWES7	0.78
UWES8	0.79
UWES9	0.65

In addition to this, we also conducted EFA using promax rotation and enforcing a three-factor structure, in order to compare the fit of the theoretical dimensionality of the UWES-9 with the one-factor solution we found in our sample. The χ2 for this model was 45,72 (df 12) (*p* < 0.001). The items did not load on their expected factors “Dedication” had 4 items (3, 4, 5, 6), “Vigor” had 2 items (1, 2), and “Absorption” had 3 items (7, 8, 9).

### Confirmatory Factor Analysis

As the EFA suggested a one-factor solution, as described above, the model was first specified with just one latent factor (Work Engagement). Standardized coefficients were used and the estimation model was maximum likelihood, since the items showed acceptable skewness and kurtosis (Table 2). Observations with missing values were excluded.

In order to also test the theoretical foundation of the UWES-9, we performed CFA with the original three subscales Vigor, Dedication and Absorption. Additionally, inspired by a previous study by Kulikowski (2019), who also tested a two-factor model, we also performed CFA using this structure.

**FIGURE 1:**
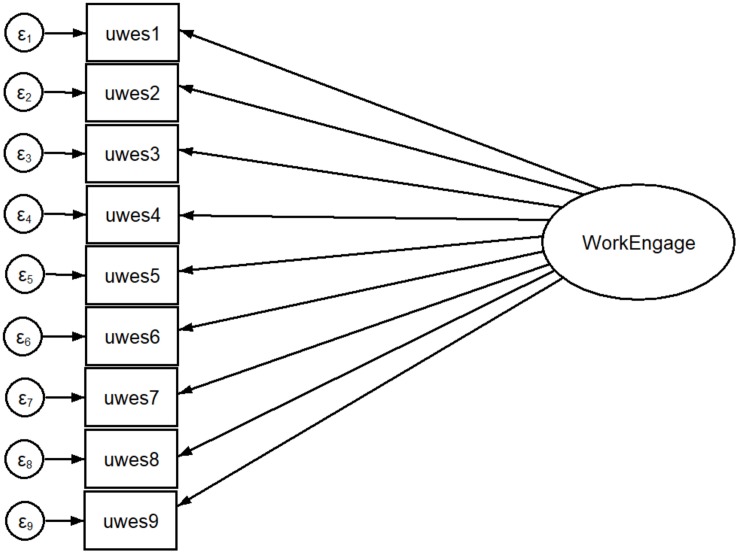
One-factor structure with maximum likelihood estimation.

**FIGURE 2:**
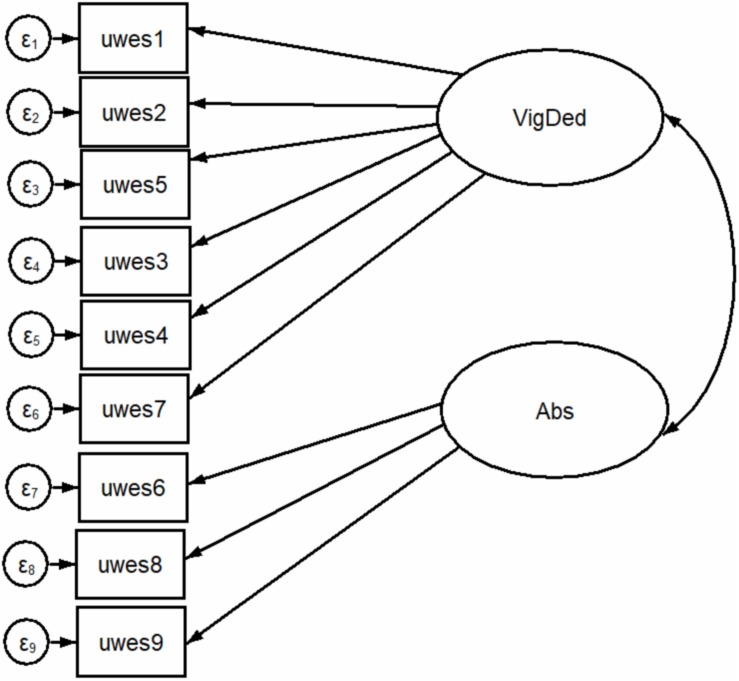
Two-factor structure with maximum likelihood estimation.

**FIGURE 3:**
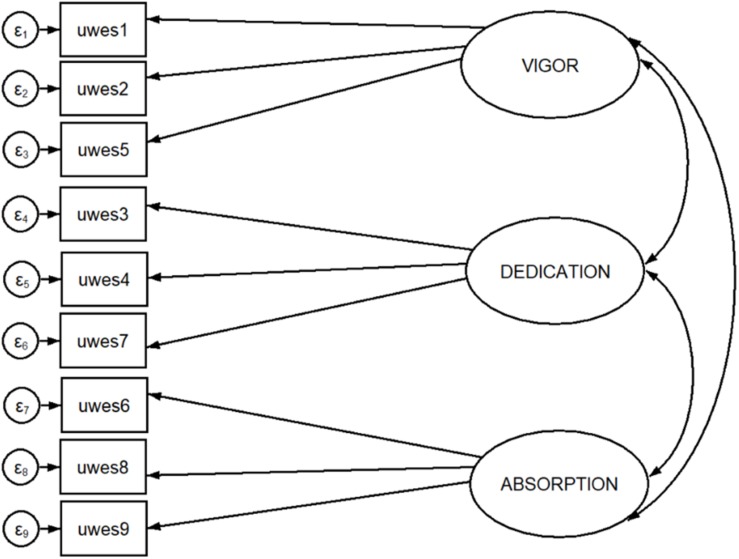
Three-factor structure with maximum likelihood estimation.

Figures 1–3 show all the attempted models.

Table 4 shows the coefficients of the hypothesized relationships, together with their *z*-values, standard errors, 95% confidence intervals and *p*-values, for all tested models.

**TABLE 4:** All models’ standardized coefficients and associated data.

**Item**	**Coefficient**	**Standard error**	***z*-value**	***p*-value**	**95% CI**
**One-factor model**					
Item 1	0.79	0.02	50.42	<0.0001	0.76; 0.82
Item 2	0.82	0.01	59.95	<0.0001	0.79; 0.85
Item 3	0.92	0.01	132.99	<0.0001	0.91; 0.94
Item 4	0.90	0.01	109.15	<0.0001	0.89; 0.92
Item 5	0.81	0.01	55.85	<0.0001	0.78; 0.83
Item 6	0.87	0.01	83.55	<0.0001	0.85; 0.89
Item 7	0.76	0.02	44.83	<0.0001	0.73; 0.80
Item 8	0.81	0.01	57.54	<0.0001	0.78; 0.84
Item9	0.69	0.02	33.19	<0.0001	0.65; 0.73
**Two-factor model^∗^**					
Item 1	0.80	0.02	36.08	<0.0001	0.75; 0.84
Item 2	0.83	0.02	42.84	<0.0001	0.79; 0.87
Item 3	0.92	0.01	80.72	<0.0001	0.89; 0.94
Item 4	0.90	0.01	67.82	<0.0001	0.87; 0.92
Item 5	0.76	0.02	31.74	<0.0001	0.72; 0.81
Item 6	0.89	0.02	56.27	<0.0001	0.86; 0.92
Item 7	0.77	0.02	32.23	<0.0001	0.72; 0.81
Item 8	0.83	0.02	40.16	<0.0001	0.79; 0.87
Item 9	0.76	0.03	28.72	<0.0001	0.71; 0.81
**Three-factor model^∗∗^**					
Item 1	0.89	0.01	81.70	<0.0001	0.87; 0.91
Item 2	0.92	0.01	93.13	<0.0001	0.90; 0.94
Item 3	0.94	0.01	147.03	<0.0001	0.93; 0.95
Item 4	0.93	0.01	128.89	<0.0001	0.91; 0.94
Item 5	0.74	0.02	36.26	<0.0001	0.70; 0.78
Item 6	0.99	0.01	80.71	<0.0001	0.86; 0.90
Item 7	0.75	0.02	42.25	<0.0001	0.72; 0.79
Item 8	0.84	0.02	60.96	<0.0001	0.81; 0.86
Item 9	0.73	0.02	36.10	<0.0001	0.69; 0.77

*^∗^Items 1, 2, 3, 4, 5, and 7 belong to the combined vigor/dedication factor. Items 6, 8, and 9 belong to the absorption factor.^∗∗^Items 1, 2, and 4 belong to the vigor factor. Items 3, 4, and 7 belong to the dedication factor. Items 6, 8, and 9 belong to the absorption factor.*

After estimating the models, goodness-of-fit statistics were obtained, as described in the section “Materials and Methods,” above. As can be seen in Table 5, none of the models showed very good fit, with RMSEA ranging between 0.181 and 0.167. Also, CFI and TLI, which should preferably be above 0.95 (Hooper et al., 2008) remained below this value for all tested models.

**TABLE 5:** Goodness-of-fit statistics for all models.

**Fit statistic**	**One-factor model**	**Two-factor model**	**Three-factor model**
Chi2 (df)	633.90 (27)	354.49 (26)	247.76 (24)
RMSEA (90% CI)	0.181 (0.169; 0.194)	0.192 (0.175; 0.192)	0.167 (0.154; 0.180)
AIC	16221.47	8246.29	8143.56
BIC	16343.70	8353.66	8258.60
CFI	0.895	0.882	0.920
TLI	0.860	0.837	0.880
SRMR	0.046	0.049	0.065

*Df, degrees of freedom; RMSEA, root mean squared error of approximation; CI, confidence interval; AIC, Akaike’s information criterion; BIC, Bayesian information criterion; CFI, comparative fit index; TLI, Tucker-Lewis index; SRMR, standardized root mean squared residual.*

## Discussion

The aim of the present study was to use exploratory and CFA to investigate the factorial structure of the UWES in a multi-occupational sample of Swedish women. The EFA seemed to mainly favor a one-factor solution, which was shown to explain over 70% of the variance.

Confirmatory factor analysis was then performed using three different models: one-factor, two-factor, and three-factor. Goodness-of-fit statistics were obtained for all models and showed that none of them showed overall good fit, with RMSEA never going below 0.167 and CFI and TLI remaining relatively low (Table 5).

As previously mentioned, a recent review of the factorial structure of the UWES showed inconclusive results, with some included studies showing best fit for a one-factor structure, some showing best fit for a three-factor structure, and some showing an equally good (or poor) fit for both (Kulikowski, 2017). This indicates a need for further research into the underlying factors impacting the factor structures in various samples.

One of the studies included in the Kulikowski review found that neither the one-factor nor the three-factor structure of the UWES-9 was a good fit for their data (Wefald et al., 2012). This used a sample similar to ours, both in terms of size (382 vs. 342) and level of education (in both samples, around 60% had a university degree or higher). The RMSEA was 0.18 and 0.16 for the one-factor and three-factor structures, in the Wefald study, almost identical to 0.181 and 0.167 for our study.

A previous study by Kulikowski (2019) has also attempted a two-factor structure, merging Dedication and Vigor into a single factor, letting Absorption constitute the second factor (Kulikowski, 2019). We attempted the same model in the present study, but in agreement with Kulikowski’s results, failed to obtain satisfactory goodness of fit.

The only previous Swedish study using the UWES used a sample consisting of 186 information technology (IT) consultants (37% women) and found that both the one-factor and three-factor structure showed similar fit, with RMSEA of 0.13 and CFI of 0.97 for both (Hallberg and Schaufeli, 2006). Although this sample was Swedish, it was different from that of the present study in other significant ways, such as gender (a majority were male) and occupation (all the participants were IT consultants, whilst ours was a multi-occupational sample), which may explain the differences in the results.

If our results are compared with those of other studies also using multi-occupational samples, several of them have, in agreement the Swedish study by Hallberg and Schaufeli (2006), found that *both* the one-factor and three-factor structures may be used. For example, this was the case for Schaufeli et al. (2006) with a very large multinational sample of 14521 individuals.

These differing results support the recommendation made by Kulikowski (2017), namely that each study using the UWES-9 should undertake their own factor analysis based on their own sample, and make a decision on which structure to use based on their own results (Kulikowski, 2017). In addition to this, and in agreement with the current study, several previous studies have found that none of the factor structures tested have shown an acceptable fit (Hallberg and Schaufeli, 2006; Wefald et al., 2012). Subsequently, researchers looking to use a measure of work engagement may wish to use another instrument in parallel with the UWES.

The present study has strengths, as well as weaknesses. The relatively large sample size of approximately 700 women made it possible to randomly divide the group into half so that both an exploratory and a CFA could be undertaken. The fact that the sample consisted exclusively of women may be seen both as a strength and as a weakness. On the one hand, it ensures that the results are not skewed by an uneven gender balance, but on the other hand our results should not be assumed to be generalizable to males. An Iranian study investigating determinants of work engagement in hospital staff found no significant effect of gender (Mahboubi et al., 2014). However, a Dutch study exploring work engagement and burnout in veterinarians found that women rated their work engagement lower than men, indicating that gender differences may vary with different occupational groups, nationalities, or other, hitherto unknown factors (Mastenbroek et al., 2014).

In addition to this, in terms of generalizability, it should be acknowledged that the sample used in the present study should be considered to represent the white-collar population, based on the higher-than-average level of education. More than 60% of the participants reported having at least 3 years of university education, whilst the national average for women between the ages of 25 and 34 is 35%, according to Statistics Sweden (Statistics Sweden, 2017). In addition to this, only Swedish-speaking girls participated. However, 21.6% had immigrated or had parents who had immigrated to Sweden, which is in line with the population in general (Statistics Sweden, 2018).

## Conclusion

The present study used a large, multi-occupational female sample to explore the factorial structure of the UWES-9. Despite indication from EFA that a one-factor structure best fit the data, we were unable to find good model fit for a one-, two-, or three-factor model using CFA. As previous studies have also failed to reach conclusive results on the optimal factor structure for the UWES-9, further research is needed in order to disentangle the possible effects of gender, nationality and occupation on work engagement. Until such data exists, researchers would be wise to conduct their own factor analysis in order to determine whether the total score, the three dimensions representing Vigor, Dedication and Absorption, or even a two-factor structure is applicable for their sample.

## Data Availability Statement

The datasets generated for this study are available on request to the corresponding author.

## Ethics Statement

This project was approved by the Regional Ethics Board (2014/401). At the time of the data collection for the present study, the participants were again asked to give their consent and reminded that their participation was voluntary, could be withdrawn any time without giving a reason, and that all information would be treated confidentially. All participants who completed the data collection were offered a cinema ticket or a department store gift voucher as thanks.

## Author Contributions

MW contributed to the conception and design of the work, performed the analyses, and drafted the manuscript. JW and ML contributed to the conception and design of the work, took part in the data collection and analyses, and revised the work critically. All authors approved the final version to be published, and agreed to be accountable for all aspects of the work in ensuring that questions related to the accuracy or integrity of any part of the work are appropriately investigated and resolved.

## Conflict of Interest

The authors declare that the research was conducted in the absence of any commercial or financial relationships that could be construed as a potential conflict of interest.

## References

[B1] Bakker A. B. Matthijs Bal P. (2010). Weekly work engagement and performance: a study among starting teachers. *J. Occup. Organ. Psychol.* 83 189–206. 10.1348/096317909X402596

[B2] Bentler P. M. (1990). Comparative fit indexes in structural models. *Psychol. Bull.* 107 238–246. 10.1037/0033-2909.107.2.238 2320703

[B3] Bollen K. A. (2014). *Structural Equations with Latent Variables.* New York, NY: Wiley.

[B4] Christian M. S. Adela S. G. Slaughter J. E. (2011). Work engagement: a quantitative review and test of its relation with task and contextual performance. *Pers. Psychol.* 64 89–136. 10.1111/j.1744-6570.2010.01203.x

[B5] IBM Corp (2016). *SPSS for Windows.* Armonk, NY: IBM Corp.

[B6] Dziuban C. D. Shirkey E. C. (1974). When is a correlation matrix appropriate for factor analysis? Some decision rules. *Psychol. Bull.* 81 358–361. 10.1037/h0036316

[B7] Gable S. L. Haidt J. (2005). What (and why) is positive psychology? *Rev. Gen. Psychol.* 9 103–110. 10.1037/1089-2680.9.2.103

[B8] González-Romá V. Schaufeli W. B. Bakker A. B. Lloret S. (2006). Burnout and work engagement: independent factors or opposite poles? *J. Vocat. Behav.* 68 165–174. 10.1016/j.jvb.2005.01.003

[B9] Hallberg U. E. Schaufeli W. B. (2006). “Same same” but different? Can work engagement be discriminated from job involvement and organizational commitment? *Eur. Psychol.* 11 119–127. 10.1027/1016-9040.11.2.119

[B10] Ho Kim W. Park J. G. Kwon B. (2017). Work engagement in South Korea. *Psychol. Rep.* 120 561–578. 10.1177/0033294117697085 28558613

[B11] Hooper D. Coughlan J. Mullen M. (2008). Structural equation modelling: guidelines for determining model fit. *Electron. J. Bus. Res. Methods* 6 53–60.

[B12] Kulikowski K. (2017). Do we all agree on how to measure work engagement? Factorial validity of Utrecht work engagement scale as a standard measurement tool – A literature review. *Int. J. Occup. Med. Environ. Health* 30 161–175. 10.13075/ijomeh.1896.00947 28366949

[B13] Kulikowski K. (2019). One, two or three dimensions of work engagement? Testing the factorial validity of the Utrecht work engagement scale on a sample of Polish employees. *Int. J. Occup. Saf. Ergon.* 25 241–249. 10.1080/10803548.2017.1371958 28849984

[B14] Leijten F. van den Heuvel S. G. van der Beek A. J. Ybema J. F. Robroek S. J. Burdorf A. (2015). ‘Associations of work-related factors and work engagement with mental and physical health: a 1-year follow-up study among older workers. *J. Occup. Rehabil.* 25 86–95. 10.1007/s10926-014-9525-6 24928413

[B15] Mahboubi M. Ghahramani F. Mohammadi M. Amani N. Mousavi S. H. Moradi F. (2014). Evaluation of work engagement and its determinants in Kermanshah hospitals staff in 2013. *Glob. J. Health Sci.* 7 170–176. 10.5539/gjhs.v7n2p170 25716395 PMC4796363

[B16] Mastenbroek N. J. Jaarsma A. D. Demerouti E. Muijtjens A. M. Scherpbier A. J. van Beukelen P. (2014). Burnout and engagement, and its predictors in young veterinary professionals: the influence of gender. *Vet. Rec.* 174:144. 10.1136/vr.101762 24306199

[B17] Mills M. Culbertson S. Fullagar C. (2012). Conceptualizing and measuring engagement: an analysis of the Utrecht work engagement scale. *J. Happiness Stud.* 13 519–545. 10.1007/s10902-011-9277-3

[B18] Nerstad C. G. L. Richardsen A. M. Martinussen M. (2010). Factorial validity of the Utrecht work engagement scale (UWES) across occupational groups in Norway. *Scand. J. Psychol.* 51 326–333. 10.1111/j.1467-9450.2009.00770.x 20015117

[B19] O’Connor B. P. (2000). SPSS and SAS programs for determining the number of components using parallel analysis and Velicer’s MAP test. *Behav. Res. Methods Instrum. Comput.* 32 396–402. 10.3758/bf03200807 11029811

[B20] Petrović I. B. Vukelić M. čizmić S. (2017). Work engagement in Serbia: psychometric properties of the Serbian version of the Utrecht work engagement scale (UWES). *Front. Psychol.* 8:1799. 10.3389/fpsyg.2017.01799 29085319 PMC5650702

[B21] Schaufeli W. (2018). Work engagement in Europe: relations with national economy, governance and culture. *Organ. Dyn.* 47 99–106.

[B22] Schaufeli W. B. Bakker A. B. Salanova M. (2006). The measurement of work engagement with a short questionnaire a cross-national study. *Educ. Psychol. Meas.* 66 701–716. 10.1177/0013164405282471

[B23] Schaufeli W. B. Salanova M. González-romá V. Bakker A. B. (2002). The measurement of engagement and burnout: a two sample confirmatory factor analytic approach. *J. Happiness Stud.* 3 71–92.

[B24] Seppälä P. Mauno S. Feldt T. Hakanen J. Kinnunen U. Tolvanen A. (2009). The construct validity of the Utrecht work engagement scale: multisample and longitudinal evidence. *J. Happiness Stud.* 10 459–481. 10.1007/s10902-008-9100-y

[B25] Shirom A. (2003). “Feeling vigorous at work? The construct of vigor and the study of positive affect in organizations,” in *Emotional and Physiological Processes and Positive Intervention Strategies (Research in Occupational Stress and Well-being*, Vol. 3 eds Perrewe P. L. Ganster D. C. (Bingley: Emerald Group Publishing Limited), 135–164. 10.1016/s1479-3555(03)03004-x

[B26] StataCorp (2015). *Stata Statistical Software: Release 14.* College Station, TX: StataCorp.

[B27] Statistics Sweden (2017). *Befolkningens Utbildning.* Available at: http://www.scb.se/uf0506 (accessed July 2, 2018).

[B28] Statistics Sweden (2018). *Folkmängd Och Befolkningsförändringar 2017.* Available at: http://www.scb.se/hitta-statistik/statistik-efter-amne/befolkning/befolkningens-sammansattning/befolkningsstatistik/pong/statistiknyhet/folkmangd-och-befolkningsforandringar-20172/ (accessed July 2, 2018).

[B29] Steiger J. H. (1990). Structural model evaluation and modification: an interval estimation approach. *Multivariate Behav. Res.* 25 173–180. 10.1207/s15327906mbr2502_4 26794479

[B30] Torp S. Grimsmo A. Hagen S. Duran A. Gudbergsson S. B. (2013). Work engagement: a practical measure for workplace health promotion? *Health Promot. Int.* 28 387–396. 10.1093/heapro/das022 22692482

[B31] Velicer W. F. (1976). Determining the number of components from the matrix of partial correlations. *Psychometrika* 41 321–327. 10.1007/bf02293557

[B32] Wefald A. J. Mills M. J. Smith M. R. Downey R. G. (2012). A comparison of three job engagement measures: examining their factorial and criterion-related validity. *Appl. Psychol. Health Well Being* 4 67–90. 10.1111/j.1758-0854.2011.01059.x 26286971

[B33] Westerberg-Jacobson J. Edlund B. Ghaderi A. (2010). A 5-year longitudinal study of the relationship between the wish to be thinner, lifestyle behaviours and disturbed eating in 9-20-year old girls. *Eur. Eat. Disord. Rev.* 18 207–219. 10.1002/erv.98 20443204

